# Enterobius vermicularis: Coming Soon to an Appendix Near You?

**DOI:** 10.7759/cureus.84894

**Published:** 2025-05-27

**Authors:** Adam Heininger, John C Densmore

**Affiliations:** 1 General Surgery, Marshfield Clinic Health System, Marshfield, USA; 2 Pediatric Surgery, Marshfield Clinic Health System, Marshfield, USA

**Keywords:** anthelmintic, appendectomy, appendicitis, appendix, enterobius vermicularis, enterocutaneous, histopathology, mebendazole, pinworms, socioeconomic

## Abstract

In underdeveloped countries, *Enterobius vermicularis* is one of the most prevalent parasitic infections of the gastrointestinal tract. *E. vermicularis’* presence within the appendix has been shown to cause symptoms that mirror classic appendicitis but frequently does not have any histological evidence of acute inflammation. Although not an unprecedented finding in developing countries, literature rarely examines the incidence in Western countries. This report presents the case of a nine-year-old adolescent girl from the Midwestern United States who showed evidence of *E. vermicularis* within an appendectomy specimen. She presented with a four-day history of abdominal pain. Laboratory values were consistent with elevated infectious markers for a parasite infection, and radiologic findings suggested acute appendicitis. However, histopathology revealed a benign appendix with intraluminal parasitic worms most consistent with *E. vermicularis.* This case serves as a reminder that, although it is more common to find evidence of *E. vermicularis* within appendectomy specimens in developing countries, it cannot be completely ruled out in developed countries also, especially in rural settings and low-resourced environments.

## Introduction

The discovery of *Enterobius vermicularis* (*E. vermicularis*, pinworm) within the pathologic specimen following appendectomy is a rare event only occasionally reported in the surgical literature. A retrospective study out of Turkey examining 3,222 appendectomies found a frequency of 0.74% of resected appendices that were associated with *E. vermicularis* [1]. Most cases are reported in developing countries, but little is known about the incidence in the United States. Additionally, the role of *E. vermicularis* as a cause of acute appendicitis has been controversial. A study by Hasan et al. showed that, of 1,150 appendectomies, *E. vermicularis* was seen in 2.7% of cases [2]. Of the 31 cases with pinworm, 24 (77.1%) revealed *E. vermicularis* without any other pathology, and only one case showed an acute inflammatory process [2]. *E. vermicularis’* presence within the appendix has been documented to cause a clinical presentation that mimics the usual symptoms of appendicitis but frequently does not have any histological evidence of acute inflammation, making this a diagnostic challenge when a patient presents with a history, physical exam, and radiologic findings equivocal for acute appendicitis.

In underdeveloped countries, *E. vermicularis* is one of the most prevalent parasitic infections of the gastrointestinal tract. Between 4% and 28% of children in the world are reported to be infected [3]. Pinworms measure approximately 10 mm in length and live mainly within the right colon, usually in the cecum. This raises the question why more appendiceal specimens are not associated with *E. vermicularis* infection if the parasites live in such proximity to the appendix?

The most common mode of infection in humans occurs via the fecal-oral route after a gravid female pinworm deposits eggs on the perianal folds. Additionally, the parasite spreads easily among family members because its eggs can remain viable for two to three weeks on clothing and bedding. Once infectious eggs are ingested, the larvae hatch within the small intestine and then migrate to the colon as adults. The adult lifespan is about two months, and the time interval from ingestion of infectious eggs to deposit of eggs by an adult female in the perianal region is about one month.

Symptoms due to *E. vermicularis* infection can range from asymptomatic to perianal pruritus. However, more serious infestations can lead to ileocolitis, enterocutaneous fistula, urinary tract infection, mesenteric abscesses, salpingitis, and appendicitis. Laboratory evaluation during the presence of *E. vermicularis* infection is associated with immunoglobulin E (IgE) and eosinophilia.

Conversely, acute appendicitis is one of the most frequent indications of an acute abdomen requiring surgery. Causes of acute appendicitis are uncertain, but the pathology demonstrates luminal obstruction of the appendix by inflamed mucosal-associated lymphatic tissue, fecalith, or stool.

Here, we present an unusual case report of a young girl from the Midwestern United States with suspected appendicitis who was found to have an *E. vermicularis* infection postoperatively. This case report highlights the clinical significance of recognizing pinworm as a differential diagnosis in low-resourced patients who have an equivocal presentation for acute appendicitis to avoid unnecessary surgical interventions. Although pediatric laparoscopic surgery is low risk, there are unforeseen stressors associated with a visit to the operating theater. Such as the financial burden placed on an already impoverished family or the mental stress placed on a young child. The objective of this case report is to highlight the importance of including parasitic infection in the differential diagnosis for abdominal pain in school-aged children, particularly those from low socioeconomic backgrounds with eosinophilia and equivocal radiological findings for acute appendicitis, which may help avoid a visit to the operating room.

## Case presentation

A nine-year-old female, with a past medical history of precocious puberty with menarche at seven years of age and no significant past surgical history, presented with a four-day history of periumbilical and suprapubic abdominal pain that had worsened over the prior day. Heating pads and ibuprofen had not provided any relief. She denied associated symptoms of fever, chills, nausea, vomiting, or issues with urination. She had been tolerating a diet but endorsed a decrease in appetite. The patient had no known medication allergies. Home medications included melatonin for sleep and an EpiPen as necessary for anaphylaxis due to bee stings. Family history and social history were noncontributory. Based on the patient's history, the initial working diagnosis was that she had early acute appendicitis. However, laboratory evaluation and imaging findings did not fully solidify this as the etiology of her symptoms. Thus, it presented a diagnostic dilemma and necessitated a discussion with the family about the role of surgical intervention.

**Figure 1:**
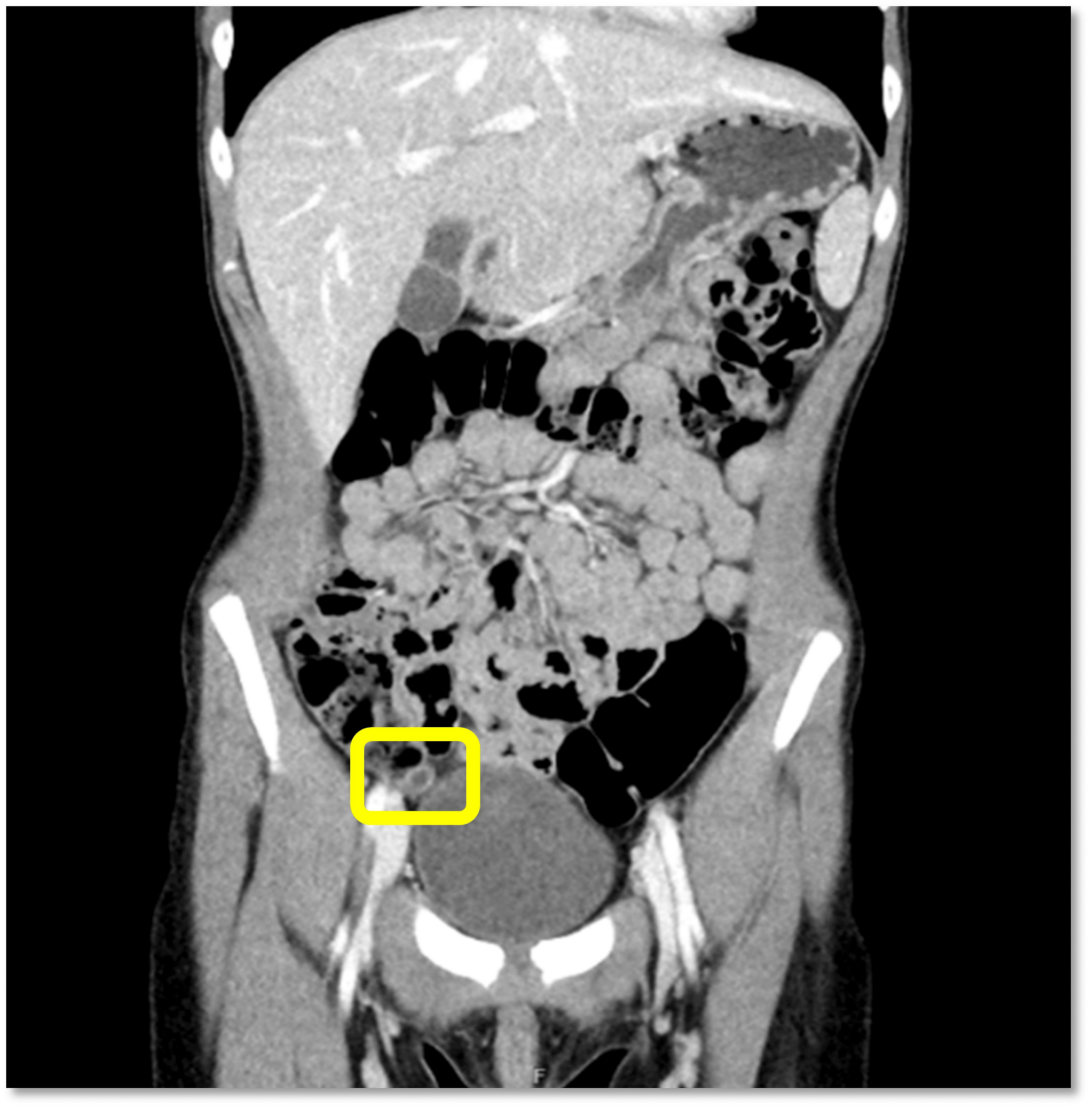
A coronal computed tomography (CT) image of the abdomen and pelvis with intravenous contrast demonstrating a mildly distended appendix. The appendix (highlighted in a yellow box) is mildly distended, measuring up to 0.7-0.8 cm in diameter, and contains both fluid and air. The appendiceal wall demonstrates borderline mucosal thickening without peri-appendiceal fat stranding or surrounding fluid collections. No intraluminal calcifications or appendicoliths are seen. Findings are consistent with early acute appendicitis.

The abdominal exam revealed tenderness in the lower abdomen consistent with her reported symptoms. Although her abdomen was soft without guarding, rebound tenderness, or peritoneal signs, her vital signs were within range for her age; she was afebrile, not tachycardic, and her oxygen saturation was 99% on room air. Laboratory investigations were significant for a 14.2% eosinophils and 0.97x10^3^/uL absolute eosinophils (Table 1). A computed tomography (CT) scan demonstrated an appendix both fluid and air-filled, with borderline distention measuring up to 0.7-0.8 cm, as well as borderline mucosal hyperplasia/thickening (Figure 1). There was no evidence of significant adjacent inflammatory fat stranding. No associated intraluminal radiodense stone or appendicolith was seen on the CT scan. These radiologic findings were equivocal and may have represented early acute appendicitis, supporting the working diagnosis of acute appendicitis and the need for surgical intervention. She was treated with 502.4 mg of acetaminophen, a 500 mL bolus of sodium chloride 0.9%, and a dose of piperacillin-tazobactam 80 mg/kg while in the emergency department.

**Table 1:** Laboratory results including C-reactive protein and complete blood count with automated differential and reference ranges. The patient's laboratory results listed underneath the 'Value' column. Notable findings include elevated eosinophils at 14.2% and an absolute eosinophil count of 0.97x10^3^/uL, which is greater than two times the upper limit of normal for the reference range. Furthermore, the inflammatory marker C-reactive protein (CRP) and white blood count (WBC) are not elevated, and the absolute neutrophil count falls within the reference range of normal. These laboratory values more consistently align with a parasite infection.

Component	Value	Reference Range
C-Reactive Protein (CRP)	<0.05	0.0-0.3 mg/dL
White Blood Cell (WBC)	6.81	4.50-17.00x10^3^/uL
Red Blood Cell (RBC)	4.73	4.00-5.60x10^6^/uL
Hemoglobin (HGB)	13.4	11.5-16.1 G/DL
Hematocrit (HCT)	38.3	33.0-47.0%
Platelets (PLT)	259	140-440x10^3^/uL
Neutrophils	34.4	%
Lymphocytes	40.5	%
Eosinophils	14.2	%
Basophils	0.9	%
Absolute Neutrophils	2.34	1.70-7.00x10^3^/uL
Absolute Lymphocytes	2.76	1.5-7.0x10^3^/uL
Absolute Eosinophils	0.97	0.00-0.40x10^3^/uL
Absolute Basophils	0.06	0.0-0.10x10^3^/uL

**Figure 2:**
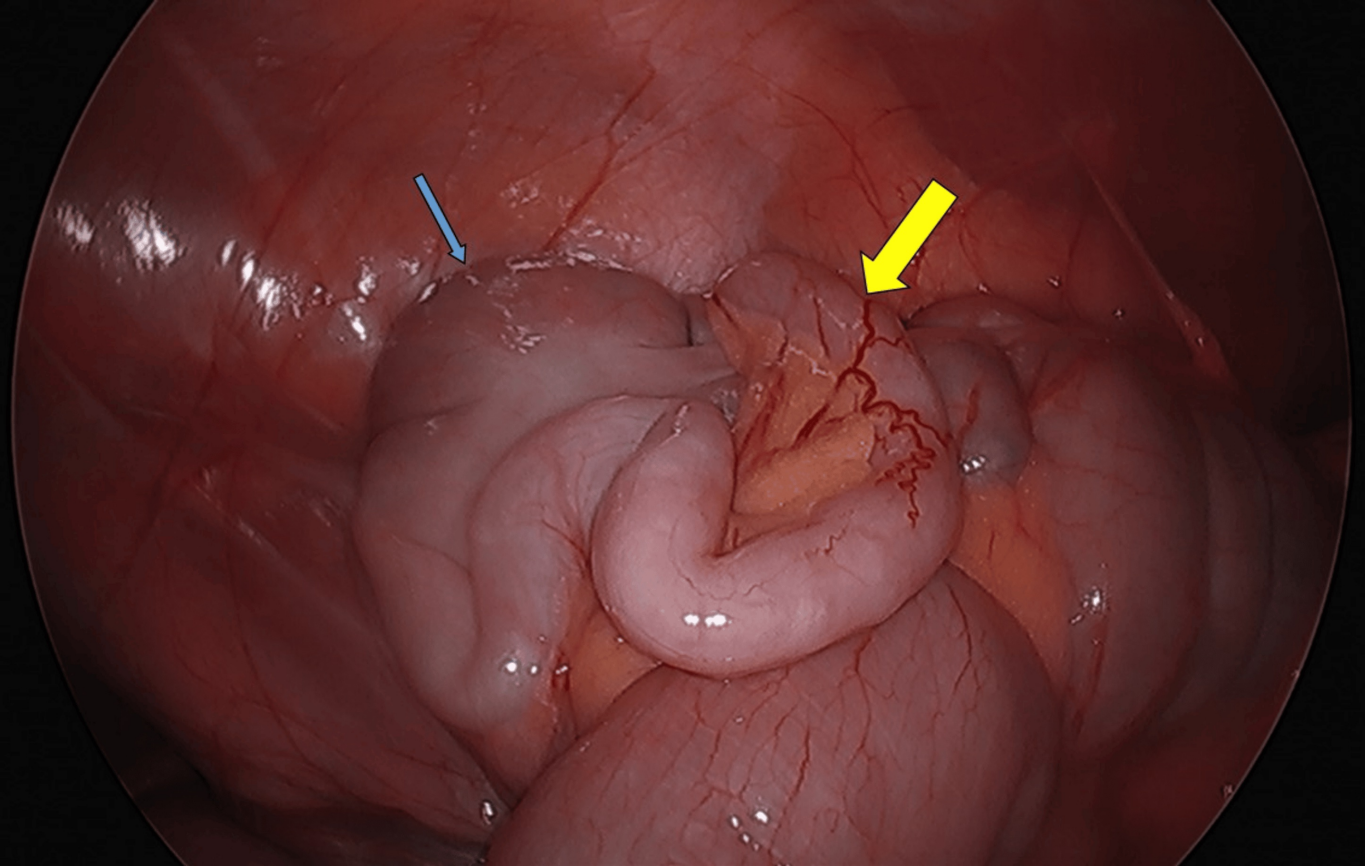
Intraoperative laparoscopic image of the cecum, appendix, and small bowel. The yellow arrow indicates the tip of the appendix, which is mildly dilated and demonstrates prominent surface vascularity (hyperemia and injections), signs consistent with early inflammation and consistent with early acute appendicitis. No evidence of appendiceal perforation or peri-appendiceal abscess is present. Minimal surrounding inflammation and the absence of seropurulent fluid in the pelvis further support a diagnosis of early acute appendicitis. The blue arrow marks a normal-appearing cecum.

**Figure 3:**
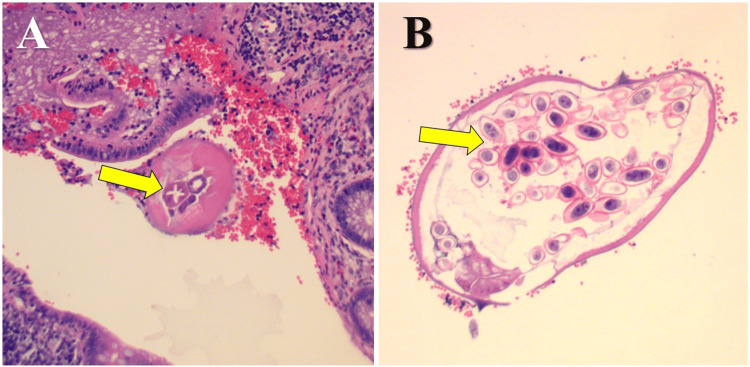
Histopathological examination revealing Enterobius vermicularis in the appendiceal lumen. Hematoxylin and eosin (H&E)-stained histopathological sections of the appendix. (A) Low-power (10x magnification) cross-section showing a transverse section of *E. vermicularis* within the appendiceal lumen. The parasite exhibits identifiable lateral algae and internal structures, including the *E. vermicularis* intestines (yellow arrow), surrounded by red blood cells and reactive lymphoid tissue. (B) High-power (40x magnification) view of a gravid *E. vermicularis* worm containing numerous oval eggs (yellow arrow) with a thick, birefringent shell and visible internal larval structures.

After a thorough discussion with the family about potential treatment options, including diagnostic laparoscopy with appendectomy, or admission for observation, under shared decision-making, it was decided to proceed to the operating room. She underwent diagnostic laparoscopy with appendectomy. Intraoperatively, the appendix was found to be dilatated at the tip with hyperemia and injections (Figure 2). There was very minimal peri-appendiceal inflammation. The appendix was clearly not perforated, and there was no seropurulent fluid within the pelvis. Again, these findings, along with the CT imaging, suggested that the patient had early acute appendicitis. However, the histopathology report revealed a benign appendix with exuberant reactive lymphoid hyperplasia and a few intraluminal parasitic worms, most consistent with *E. vermicularis* (Figure 3) on postoperative day two. Contrary to the CT scan and intraoperative findings, the histopathology report did not show evidence of acute appendicitis. Rather interestingly, the histopathology report demonstrated evidence of *E. vermicularis* infection. Therefore, further medical treatment was necessary. She was contacted at home and appropriately treated with a course of mebendazole. Her recovery was assessed on postoperative day five, given this new diagnosis, at which time she was recovering well and remained asymptomatic. It was recommended to the patient’s family to be tested and treated as well, which they were agreeable with.

## Discussion

Acute appendicitis is one of the most common surgical emergencies and spans a broad clinical spectrum from nonspecific abnormalities to appendiceal perforations. The differential diagnosis when evaluating a patient with symptoms consistent with appendicitis is quite large. Opportunities to differentiate rare causes of right lower quadrant pain from the large clinical burden of appendicitis can be fleeting. In retrospect, the prolonged four-day history without significant rapid progression of illness, the presence of eosinophilia, and relatively mild radiologic findings were clues that this patient did not have typical appendicitis. Laboratory evaluation consistent with typical acute appendicitis would more likely show a leukocytosis with elevated neutrophils rather than eosinophilia as seen in this case. Taking this into consideration, a preoperative alternative diagnosis might have altered the management and the patient's outcome.

On CT imaging, a normal appendix typically measures less than 0.6 cm in diameter and has a thin wall, usually less than 2 mm. The appendix seen on this CT scan nearly satisfied some of the criteria to be considered abnormal, possibly indicating early acute appendicitis. While the gold standard imaging modality for acute appendicitis is CT imaging, the borderline findings limit its ability to more conclusively define the patient's etiology of abdominal pain. Additionally, there are no imaging criteria to distinguish appendicitis from parasitic infections in pediatric patients. Diagnostic imaging is limited in the diagnosis of pinworm infection, which is diagnosed based on a patient's history, usually noting the classic symptom of perianal itching and eosinophilia on laboratory testing, as well as the Scotch tape test. Given the safety of pediatric laparoscopy and the risk of missed appendicitis, the decision to proceed surgically was reasonable. Intra-operatively, the appendix was noted to have hyperemia and increased vascularity, again signs indicating early acute appendicitis. However, both CT imaging and intra-operative findings were inconsistent with what might be expected in a patient presenting with four days of abdominal pain due to appendicitis. Additionally, a closer interpretation of the discordant finding of eosinophilia on laboratory testing might have prompted earlier diagnostic evaluation and consideration for other potential etiologies, likely parasitic in origin, of abdominal pain in a nine-year-old child.

*E. vermicularis* eggs hatch within the stomach, and the larvae migrate to the cecum, where they develop into adult pinworms. It is frequently observed in school-aged children, and in closely cohabitating cohorts, resulting in perianal itching, vaginal itching, and sleep disturbance due to itching when symptoms do occur. In the United States, 20-42 million people are affected by pinworm infections, and a billion people are impacted worldwide [2]. Given the disease burden and cecal location of larval maturation, it is curious that *E. vermicularis*-induced appendectomies are not more common. Anthelmintic therapies for pinworm infection are associated with cure rates of 90-100% in some studies [4] and include mebendazole, albendazole, and pyrantel pamoate.

When inflammatory changes in the appendix are found in cases of *E. vermicularis*, the pathogenic contribution of the parasite is uncertain, and its presence may be incidental. Most studies report a low incidence of inflammatory changes seen on pathological examination of appendiceal pinworms, with rates of inflammation ranging from 13% to 37% [5]. Yildirim et al. reported a mean age of 38 years in patients who underwent appendectomy for acute appendicitis, with pathology revealing *E. vermicularis* [6]. Worldwide, there is a 0.2-41.8% reported incidence of *E. vermicularis* in those with symptoms of appendicitis [5].

The case described here, of an uninflamed appendix with incidental *E. vermicularis*, aligns with the most common findings in the literature. Despite the high prevalence of infection, *E. vermicularis* remains largely asymptomatic and is a rare histopathological finding in appendectomy specimens, particularly in Western countries. However, consideration of age, social cohort, and socioeconomic status may increase suspicion for *E. vermicularis* in the differential diagnosis for right lower quadrant pain. Subtle laboratory findings of eosinophilia and an equivocal radiological scan can assist in differentiating a parasitic infection from typical acute appendicitis to avoid potential misdiagnosis or unwarranted surgical intervention.

This case highlights the importance of tailoring the differential not only to the clinical presentation but also to the patient's background. The patient resided in a Midwestern US city with a poverty rate of 12.5%, below-average educational attainment, and a median household income approximately $20,000 lower than the national median, per the 2023 United States Census Bureau. Agriculture plays a major role in the local economy - another factor associated with higher parasitic burden and reduced healthcare access.

A few unanswered questions remain. Firstly, what is the association between pinworm and appendiceal inflammation? This question requires further investigation. However, there is a high chance that the appendix may appear benign on pathology based on the literature review and, similarly, as in this case. Secondly, what is the true incidence of pinworm found in appendiceal specimens within the United States? Although it may not be high on the differential diagnosis of a possible cause of abdominal pain in Western countries, pinworm should be considered in those presenting from a low socioeconomic background. This case report highlights the importance of tailoring the potential differential diagnoses by also factoring in the patient’s socioeconomic status.

## Conclusions

*E. vermicularis* is a rare but notable incidental finding in appendectomy specimens, yet its role in causing acute appendicitis remains uncertain. The presented case highlights the importance of including parasitic infection in the differential diagnosis for abdominal pain in school-aged children, particularly those from low socioeconomic backgrounds with eosinophilia and equivocal radiological findings for acute appendicitis. A closer evaluation of the major discordant finding of eosinophilia may have been the diagnostic clue to other potential causes of her symptomology and further diagnostic workup. While the parasite’s contribution to acute appendicitis is still debated, awareness of such atypical causes may refine clinical judgment to prevent undue surgical intervention and adopt conservative management in similar clinical scenarios. However, the opportunity to avoid unnecessary appendectomy remains low, given the low risk of surgery and the risks of delayed recognition of appendicitis. However, the rare nature of finding pinworm in an appendectomy specimen described in a single case report limits the generalizability of such recommendations to the broader population. Further research is needed to clarify the true burden and significance of *E. vermicularis* in Western surgical pathology.
